# Animal type outweighs growth stage in shaping the active antibiotic resistome in livestock manure

**DOI:** 10.3389/fmicb.2026.1898413

**Published:** 2026-09-09

**Authors:** Junmiao Li, Lang Ran, Shuyuan Xue, Yuepeng Sun

**Affiliations:** 1 School of Ecology and Environment, Inner Mongolia University, Hohhot, China; 2 Inner Mongolia Academy of Agricultural and Animal Husbandry Sciences, Hohhot, China

**Keywords:** active resistome, animal type, antibiotic resistance genes, growth stage, livestock manure, metagenomics, metatranscriptomics

## Abstract

Animal manure is a major reservoir of antibiotic resistance genes (ARGs), yet the factors governing their composition and activity remain insufficiently understood. In this study, metagenomics, metatranscriptomics, and quantitative PCR (qPCR) were integrated to characterize microbial communities and resistome profiles in manure from five animal types across multiple growth stages. Results demonstrated that animal type was the dominant factor shaping both microbial community structure and ARG composition at DNA and RNA levels, whereas growth stage did not significantly influence overall community structure. Metagenomic analysis identified 944 ARG subtypes, predominantly associated with tetracycline, multidrug, and macrolide–lincosamide–streptogramin (MLS) resistance. Metatranscriptomic analysis further revealed 579 actively transcribed ARG subtypes, among which 29 [e.g., *tet*(W), *sul*1, and *erm*(B)] were consistently detected at both DNA and RNA levels, indicating their active nature. Among tested animal types, layer litter showed the highest ARG number at the DNA level, with qPCR results further revealing significantly higher abundances of specific ARGs, including *bla*_*TEM*_ and *erm*(B). Although growth stage had limited influence on overall resistome composition, stage-dependent variations in ARG abundance were observed, with higher levels during early-life and juvenile stages. Procrustes analysis revealed strong correlations between microbial community composition and resistome profiles in both DNA and RNA levels, suggesting that microbiome structure plays a key role in shaping ARG dynamics and activity. Overall, animal type was the dominant measured correlate of resistome composition, whereas growth stage was associated mainly with variation in selected ARG abundances. These observational findings require validation in multi-farm longitudinal studies before management or environmental-risk conclusions are drawn.

## 1 Introduction

Since the early 21st century, there has been a dramatic increase in livestock and poultry breeding globally. According to FAOSTAT statistics from the Food and Agriculture Organization, the global populations of chickens increased from 14.7 billion to 33.1 billion during 2000–2020, representing increases of 120% (Mottet et al., 2017; Podhora et al., 2013). This expansion has been accompanied by the generation of livestock manure and poultry litter. Livestock manure and poultry litters, as nutrient-rich organic resources, play a positive role in enhancing soil fertility and crop productivity. However, antibiotics are generally overused or abused for the purposes of treating or preventing animal diseases and improving economic benefits by accelerating animal growth (Matheou et al., 2025; Wang et al., 2024). The overuse of antibiotics has led to severe consequences. A substantial proportion of veterinary antibiotics administered to animals is excreted into the surrounding environment via livestock excreta in an unmetabolized form (Feinman and Matheson, 1978). Antibiotics can exert selective pressure for the dissemination and prevalence of antibiotic resistant bacteria (ARB) and genes (ARGs) in soil-plant system (Liu B. et al., 2025; Liu L. et al., 2025). The propagation of ARGs poses a risk of crop contamination when animal manure is routinely applied to farmland as fertilizer (Muhammad et al., 2020; Sorinolu et al., 2021).

While livestock manures and poultry litters could be organic fertilizers if they were treated appropriately (Kadam et al., 2024), ARGs in treated manure or litter remains a concern. Livestock manure acts as a major reservoir for ARGs. Land application of organic composts contributes to the proliferation and dissemination of ARGs in soil, which can then be transferred to plant-associated microbiomes via horizontal gene transfer (HGT) (Ke et al., 2026). Beef cattle manure application resulted in the enrichment of ARGs in soil and facilitated their transmission from soil to lettuce microbiomes (Sun et al., 2025). Notably, the abundance and composition of ARGs can vary among manure types. One study indicated that chicken litters displayed higher diversity and greater abundance of ARGs compared to swine manure, even though antibiotic usage in swine farming was more intensive than that in chicken farming (Chen et al., 2019). Another study demonstrated that composts from monogastric livestock manure (e.g., chickens and pigs) harbored significantly higher abundances of ARGs than those from ruminant manure (e.g., sheep and cattle) (Wang et al., 2024). Within the same animal species, variations in ARG abundance have also been observed among different production types. For instance, layer litter exhibited higher ARG abundance than that in broiler chicken litter (Gu et al., 2020; Pham et al., 2024; Rothrock et al., 2021). Moreover, differences in the profiles of ARGs were observed in manure from the same animal species during different growth phases (Gu et al., 2020; Liu S. et al., 2025). For instance, sow manure exhibited higher ARG abundance than piglet/fattening pig manure (Liu B. et al., 2025). This phenomenon is likely attributable to variations in feeding patterns and animal ages (Liu B. et al., 2025). Feeding patterns influence ARG abundance and composition by altering management-related selective pressures, including feed formulation and antibiotic use, in animal production systems (He et al., 2020; Zhao et al., 2025). Animal age captures the cumulative and successional effects of growth-associated changes in fecal environments, resulting in stage-specific ARG dynamics (He et al., 2020). However, most existing studies have focused on a narrow subset of livestock manure types or individual growth stage, often treating manure resistomes as static endpoints rather than dynamic systems shaped by host development. In addition, information about the transcriptionally active ARGs in manure is very limited in previous studies. Most recent studies have focused primarily on DNA-level resistome characterization or abundance dynamics (Qiu et al., 2022; Xu et al., 2023), whereas only a few have incorporated metatranscriptomic approaches to assess ARG expression and functional activity in livestock-associated environments (Sun et al., 2024). Consequently, how ARG abundance, diversity, and transcriptional activity vary across animal growth stages in relation to microbiome succession and management practices remains insufficiently resolved.

This study aims to investigate whether animal type and growth stage are key factors driving the composition and activity of ARGs in livestock manure by integrating qPCR, metagenomics, and metatranscriptomics. We propose the following hypotheses: (1) the abundance, diversity, and composition of manure resistomes are associated with animal type and growth stage; (2) both genomic and transcriptomic compositions of resistome were associated with microbial community structure. The findings of this study offer evidence of the dynamics of ARGs at both DNA and RNA level and across growth stages, providing a theoretical basis for revealing propagation and control of ARGs in livestock wastes.

## 2 Materials and methods

### 2.1 Manure sample collection

Fresh manure samples were collected from five commercial farms in Hohhot, Inner Mongolia, including one pig farm, one layer farm, one sheep farm, one dairy farm, and one beef cattle farm. All sampled farms were commercial-scale operations with herd or flock sizes exceeding 1,000 individuals. At each farm, animals at different growth stages were housed indoors separately. Antibiotics were used only in limited amounts for therapeutic purposes. Samples from pigs, layers, sheep, and dairy cattle covered four growth stages: early-life, juvenile, rearing, and adult stages. The early-life stage referred to the earliest sampled growth stages for each animal type, including suckling offspring in mammals and layer chicks within approximately 1 week after hatching (Chalvon-Demersay et al., 2021; Du et al., 2023; Prunier et al., 2020), and was used solely as an analytical category without implying physiological equivalence among types. Manure samples of beef cattle were collected at three growth stages only: early-life, rearing, and adult. Samples were collected directly after defecation using sterile tools without bedding material. Three biological replicates were collected from several animals within the same pen for each animal type and growth stage. All manure samples were immediately placed on dry ice for low-temperature transport after collection. Upon arrival at the laboratory, they were transferred to −20 °C for storage until DNA and RNA extraction.

### 2.2 Total DNA and RNA extraction

Total DNA extraction was performed using the FastDNA*™* Spin Kit for Soil (Cat. No. 6560200, MP Biomedicals, Irvine, CA, USA) following the manufacturer’s protocols. Total RNA extraction was conducted with the Quick-RNA*^TM^* Fecal/Soil Microbe MicroPrep Kit (Cat. No. R2040, Zymo Research, Orange County, CA). The quality and concentration of extracted DNA and RNA were analyzed using a NanoDrop ND-2000c spectrophotometer (Thermo Fisher Scientific, Waltham, MA).

### 2.3 qPCR

Twelve ARGs were selected because they were often detected in animal manure (Amador et al., 2019; Dandachi et al., 2018; Yang et al., 2019). These ARGs include genes encoding resistance to tetracyclines [*tet*(Q), *tet*(W), *tet*(X), *tet*(M)], sulfonamides (*sul*1, *sul*2), β-lactams (*bla*_TEM_), macrolides [*erm*(B), *erm*(F)], quinolones (*qnr*A, *qnr*B), and polymyxins (*mcr*-1). The 16S rRNA gene was concurrently amplified to normalize ARG abundance.

Each 20 μL reaction comprised 10 μL ChamQ Universal SYBR qPCR Master Mix (Cat. No. Q711-02. Vazyme Biotech Co., Ltd., Nanjing, China), 1 μL of each forward and reverse primer, 1 μL DNA template, and 7 μL RNase-free water. All qPCR reactions were run in triplicate on a QuantStudio 3 Real-Time PCR System (Thermo Fisher Scientific, Waltham, MA). The thermal cycling conditions for *tet*(M) amplification by qPCR were as follows: pre-denaturation at 95°C for 2 min, followed by 40 cycles of amplification, each consisting of denaturation at 95°C for 15 s, annealing at 51.9°C for 20 s, and extension at 72°C for 20 s. Immediately after amplification, a melting curve analysis was performed from 60 to 95 °C with a ramp rate of 1.6° C/s to verify product specificity. The amplification conditions for the remaining ARGs and 16S rRNA gene were identical except for the annealing temperatures, as detailed in Supplementary Table 1. The absolute gene copy numbers were standardized using a series of 10-fold dilutions (10^1^–10^9^ copies μL^–1^) of synthetic DNA oligos. All qPCR runs had an amplification efficiency of 99.2%–109.6% and R^2^ > 0.998.

### 2.4 Metagenomic and metatranscriptome sequencing

For metagenomic and metatranscriptomic sequencing, DNA and RNA extracts from three biological replicates were pooled to generate composite samples for each animal type and growth stage. Manure DNA was subjected to 150 bp paired-end sequencing on the Illumina NovaSeq 6000 platform (Illumina, San Diego, CA, USA). For metatranscriptomic sequencing, ribosomal RNA was depleted from total RNA, followed by reverse transcription into cDNA using TruSeq Stranded mRNA LT Sample Prep Kit (Cat. No. RS-122-2101, Illumina, San Diego, CA).

Raw data were processed using Fastp (v0.20.0) with the following filtering criteria: (1) adapter overlap ≥ 3 bp and mismatch rate > 20%; (2) sequences failing sliding-window quality checks; (3) reads shorter than 50 bp; (4) reads containing ambiguous bases (N). Ribodetector (v0.3.1) was applied to the metatranscriptomic data to remove 16S rRNA sequences, using a minimum read length of 100 bp, the “rrna” detection model. After quality control, the metagenomic datasets generated 80.5 Gb of clean data, comprising 68,588,411 high-quality reads, while the metatranscriptomic datasets produced 20.73 Gb with 69,645,106 high-quality reads.

### 2.5 Antibiotic resistance genes analysis

ARGs on DNA (metagenomics) and RNA reads (metatranscriptomics) filtering by Fastp were identified using ARG_OAP3 (v3.2.4) with Stage One alignment against the SARG v3.2 database (e-value cutoff e^–10^, minimum sequence identity 45%) and Stage Two BLAST refinement (e-value cutoff e^–7^, minimum identity 80%, minimum query coverage 75%, minimum amino acid length 25) (Yin et al., 2023). ARG abundance was calculated as ARG copy per copy of 16S rRNA gene by normalizing ARG-like read counts to ARG reference length and then scaling by the length-normalized 16S rRNA gene reads (Equation 1) (Yang et al., 2016):


Abundance= (1)

$$\overset{\text{n}}{\underset{1}{\sum }}\frac{{\text{N}}_{\text{ARG}-\text{like}\,\text{sequence}}\times {\text{L}}_{\text{reads}}/{\text{L}}_{\mathrm{ARG}\,\mathrm{reference}\,\mathrm{sequence}}}{{\text{N}}_{16\,S\,\mathrm{sequence}}\times {\text{L}}_{\text{reads}}/{\text{L}}_{16\,S\,\mathrm{sequence}}}$$

where N_*ARG*−*likesequence*_ is the number of ARG-like sequences annotated to a specific ARG reference sequence; L_reads_ is the read length; L_*ARGreferencesequence*_ is the nucleotide length of that ARG reference sequence, N_16Ssequence_ is the number of 16S rRNA gene reads, and L_16Ssequence_ = 1432 bp. This 16S-normalized metric is widely used to make ARG quantification comparable across samples with different prokaryotic DNA fractions.

The quality-filtered data were also assembled using MEGAHIT (v1.2.9) with default parameters (Li et al., 2015), followed by open reading frames (ORFs) prediction from assembled contigs with Prodigal (v2.6.3) with default parameters. Gene dereplication was conducted via CD-HIT (v4.8.1) at 90% sequence identity(Fu et al., 2012). Non-redundant genes were functionally annotated against the Comprehensive Antibiotic Resistance Database (CARD) database using DIAMOND (v2.0.11) with an e-value cutoff of 1e^–5^ and coverage ≥ 80% (Liu S. et al., 2025). Based on the functional annotation results, the number of contigs corresponding to each ARG was determined. The relative abundance of individual ARG was calculated by normalizing the contig count of a given ARG to the total number of contigs annotated as ARGs (Equation 2).


$$\mathrm{Relative}\,\mathrm{abundance}=\overset{\text{m}}{\underset{1}{\sum }}\frac{{\text{C}}_{\mathrm{ARG}-\mathrm{like}\,\mathrm{contigs}}}{{\text{C}}_{\text{total}\,\mathrm{contigs}}}$$ (2)

Where C_*ARG*−*likecontigs*_ represents the number of contigs annotated as ARG-like sequences, C_totalcontigs_ denotes the total number of contigs annotated as ARGs, and m is the total number of detected ARGs.

To compare the breadth of ARG subtype detection between the metagenomic and metatranscriptomic datasets, transcriptional diversity coverage was calculated for each animal type as the number of ARG subtypes detected by metatranscriptomics divided by the number detected by metagenomics and multiplied by 100%.

### 2.6 Taxonomic annotation

After quality control with Fastp, taxonomic profiling of microbial communities was performed using MetaPhlAn (v4.4.1) with the Species-level Genome Bins (SGBs) database with default parameters(Wood and Salzberg, 2014).

### 2.7 Statistical analysis and visualization

Data visualization was performed using Origin 2021 and the *ggplot2* package in R (v 4.4.3). Statistical significance was determined at *p* < 0.05. ARG abundance data were log10-transformed before statistical analysis to improve data distribution and variance homogeneity. Effects of animal type and growth stage on ARG abundances were evaluated using two-way ANOVA in SPSS (v27.0). Tukey’s HSD test was used for pairwise comparisons, and Benjamini–Hochberg FDR correction was applied across the 12 target ARGs. Because the adjusted results did not change the conclusions, raw *p*-values are reported in Supplementary Table 6 and S7. To assess overall community composition, principal coordinates analysis (PCoA) based on Bray–Curtis dissimilarity matrices was performed in R (v4.4.3) using relative abundances at the phylum, genus, and ARG subtype levels. PERMANOVA with 999 permutations was conducted to evaluate the effects of animal type and growth stage on microbial community and resistome composition. Procrustes analysis based on the first two PCoA axes was performed using the protest function in the vegan package with 999 permutations to assess the concordance between microbial and resistome ordinations at both DNA and RNA levels.

## 3 Results

### 3.1 Microbial composition in animal manures

At the phylum level, metagenomic sequencing revealed that Firmicutes, Bacteroidota, Pseudomonadota, and Actinomycetota were the predominant phyla across manure samples. This result was consistent with previous observations in the microbiomes of herbivorous and omnivorous animal manures (Wang et al., 2023).

**FIGURE 1:**
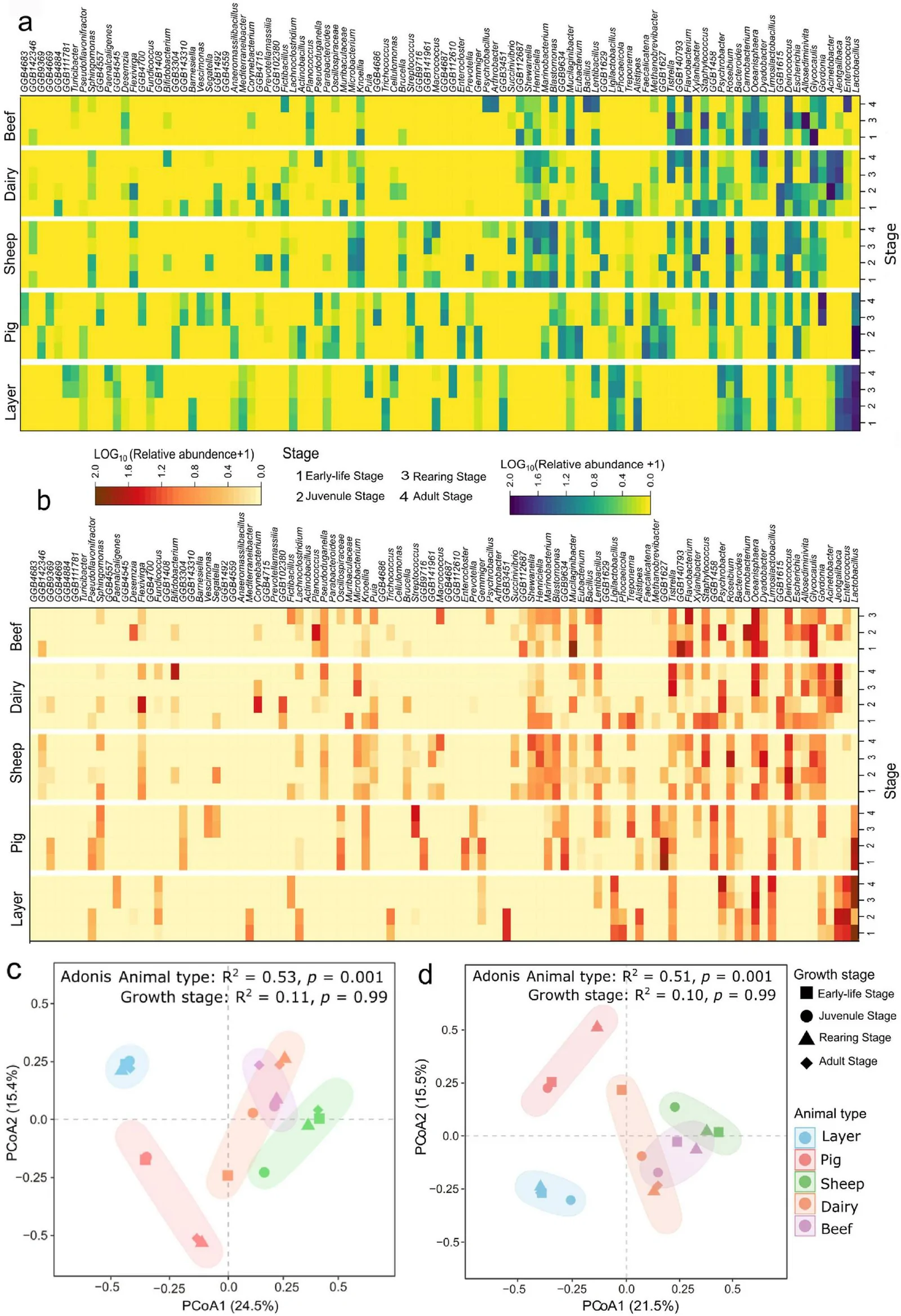
Microbial communities in livestock manure at the DNA [**(a)**, TOP100 genera] and RNA levels [**(b)**, TOP100 genera]. Principal coordinates analysis (PCoA) plots depicting the Bray–Curtis dissimilarity matrices of microbial community compositions at the genus level at the DNA **(c)** and RNA levels **(d).** Ellipses represent 95% confidence intervals according to animal type. Dairy and beef represent dairy cattle and beef cattle, respectively. Panels c and d share the same legend.

At the genus level, *Enterococcus*, *Jeotgalibaca*, and *Lactobacillus* were the dominant genera across all manures (Figure 1a). *Enterococcus* and *Lactobacillus* were significantly enriched in layer litter. *Lactobacillus* was also enriched in pig manure. *Jeotgalibaca* showed notable enrichment in both layer and dairy cattle manures. *Acinetobacter* was primarily detected and enriched in dairy cattle manure, and *Gordonia* exhibited a clear dominance in pig manure. These results demonstrated differences in the dominant bacterial genera across various animal types.

*Escherichia* was enriched during the early-life and juvenile stages across layer, pig, and dairy cattle manure, and decreased during the rearing and adult stages (Figure 1a). In dairy cattle manure, *Jeotgalibaca* abundance increased from 0.1% in the early-life to 13.33% in the rearing stage. In layer litter, abundance of *Jeotgalibaca* decreased from 8.15% in the early-life to 4.15% in the rearing stage. Taken together, the relative abundances of *Escherichia* and *Jeotgalibaca* vary with growth stages and animal types.

PCoA was conducted to examine the beta diversity among the microbiomes of various animal types and growth stages using the Bray-Curtis dissimilarity distance calculated at the genus level. The effects of animal types and growth stages on the microbial communities at the DNA level were examined (Figure 1c). The two principal coordinates captured 24.5% and 15.4% of the total variations, respectively. Manure from the same animal type clustered tightly, while manure from different animal types showed clear separation. Adonis confirmed a statistically significant difference among animal types (R^2^ = 0.53, *p* = 0.001), whereas no significant difference was observed among growth stages (R^2^ = 0.11, *p* = 0.99). These findings highlighted that animal type rather than growth stage was the major factor shaping the microbial community structure in manure.

Metatranscriptomic analysis was further employed to explore the activity of manure microbiota (i.e., transcriptionally active genera) (Figure 1b). The main transcriptionally active genera were *Acinetobacter*, *Dyadobacter*, *Enterococcus*, *Flavobacterium*, *Gordonia*, *Jeotgalibaca*, *Lactobacillus*, *Limosilactobacillus*, *Oceanisphaera* and *Roseibium*. Several genera exhibited transcriptional activity primarily in specific animal types. *Acinetobacter* showed transcriptional activity mainly in dairy cattle manure (0.43%–3.61%) and layer litter (0%–0.51%). Gordonia was transcriptionally active predominantly in pig manure (0%–5.88%). *Blastomonas* (3.55%–6.99%) and *Roseibium* (1.13%–19.54%) maintained sustained transcriptional activity in sheep manure across all growth stages. Together, these results showed that animal type determined the microbial communities at transcriptional level.

*Enterococcus*, *Limosilactobacillus*, and *Lactobacillus* showed strong transcriptional activity in monogastric animals. In pig manure, *Lactobacillus* and *Limosilactobacillus* exhibited higher transcriptional activity during the early-life stage with reduced activity toward adult stage, whereas *Enterococcus* remained transcriptionally active across growth stages. In layer litter, *Enterococcus* and *Limosilactobacillus* showed elevated transcriptional activity during the juvenile stage. *Jeotgalibaca* also displayed high transcriptional activity, peaking in the juvenile stage of layer litter and remaining detectable across growth stages in dairy cattle manure. Among ruminants, *Flavobacterium* and *Oceanisphaera* showed strong transcriptional activity in dairy cattle manure, with increased activity from early-life to adult stages, while *Dyadobacter* exhibited growth-stage-associated variation in beef cattle manure. Together, the findings of this study showed that growth stage influenced the transcriptional activity of manure microbiota, with *Lactobacillus*, *Limosilactobacillus*, *Enterococcus* being highly active in monogastric animals, whereas in ruminants, genera such as *Flavobacterium* and *Oceanisphaera* showed strong activity, with transcriptional levels varying across growth stages.

We examined the microbiomes across different animal types and growth stages at the RNA level (Figure 1d). Results showed that 21.5% and 15.5% of variation could be explained by PCo1 and PCo2, respectively. Adonis confirmed a statistically significant difference for animal types (R^2^ = 0.51, *p* = 0.001) but not for growth stages (R^2^ = 0.10, *p* = 0.99). In summary, the overall transcriptional profiles depended more on animal type than on growth stage.

### 3.2 Resistome composition

**FIGURE 2:**
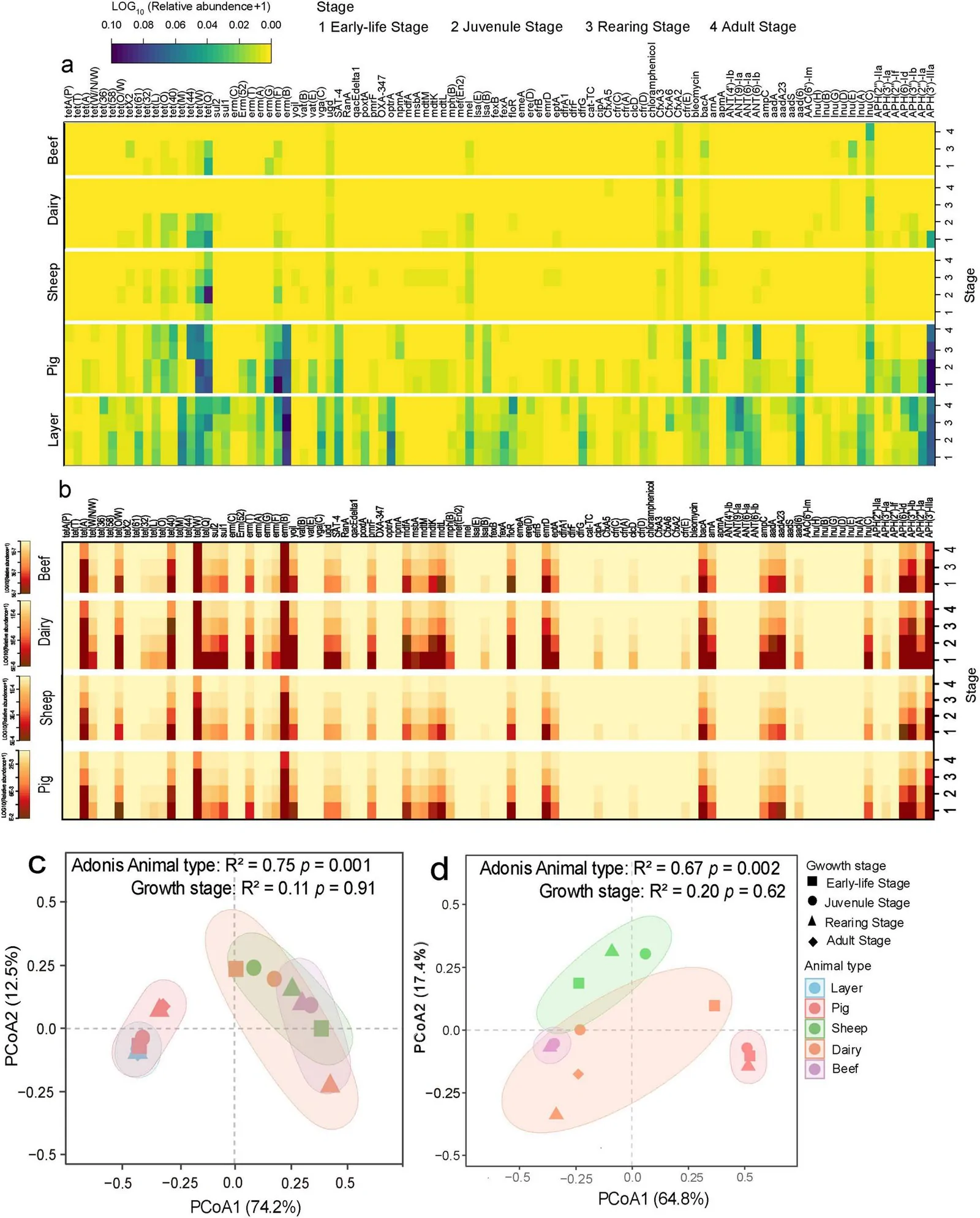
Antibiotic resistomes in livestock manure at the DNA [**(a)**, TOP100 ARG subtypes] and RNA levels [**(b)**, TOP100 ARG subtypes]. Layer samples were excluded from the RNA-level heatmap because read-based ARG-OAP analysis did not yield reliable profiles for this animal type. Principal coordinates analysis (PCoA) plots depicting the Bray–Curtis dissimilarity matrices of ARG compositions at the DNA **(c)** and RNA levels **(d)**. Ellipses represent 95% confidence intervals according to animal type. Dairy and beef represent dairy cattle and beef cattle, respectively. Panels c and d share the same legend.

To characterize the resistome profiles of fecal samples across the five animal types, integrated metagenomic and metatranscriptomic analyses were performed. Metagenomic sequencing identified a total of 944 ARG subtypes conferring resistance mainly to macrolide-lincosamide-streptogramin (MLS), tetracycline and aminoglycoside (Supplementary Figure 1). The top 100 ARG subtypes that were shared among all five animal types were visualized (Figure 2a).

Metagenomic analysis identified *tet*(Q), *lnu*(C), *tet*(W), and *aph(3’)*-IIIa as the predominant ARG subtypes (Supplementary Figure 2). Abundances of *tet*(O), *tet*(W), *tet*(Q), and *erm*(F) differed significantly among animal types, with higher levels in layer and pig manure than in sheep, dairy cattle, and beef cattle manure (Figure 2a). Across growth stages, *tet*(O) and *tet*(W) abundances generally decreased from juvenile to adult stage, whereas *tet*(Q) and *erm*(F) abundances increased from early-life to rearing stage and then declined toward adult stage. These findings indicated that both animal type and growth stage influenced the abundance of these ARG subtypes in livestock manure and layer litter.

Metatranscriptomics detected 579 active ARG subtypes (mainly *tet*(W), *tet*(Q), *lnu*(C), *erm*(B), *ugd*, Supplementary Figure 2), with main resistance types of aminoglycosides, tetracycline and MLS (Supplementary Figure 1). Top 100 ARGs identified by both metagenomics and metatranscriptomics were visualized in a heatmap (Figure 2b). This analysis revealed 29 ARG subtypes that were detected in both metagenomic and metatranscriptomic datasets [e.g., *tet*(W), *tet*(Q), *sul*2, *sul*1, *erm*(F), and *erm*(B)], verifying the transcriptional activity of these ARGs.

Due to technical limitations, reliable read-based ARG profiles could not be generated from the layer metatranscriptomic datasets using ARG-OAP. Therefore, layer litter were excluded from the comparative heatmap based on read-level annotation. Among the animal types with valid ARG-OAP metatranscriptomic profiles, pig manure exhibited the highest relative abundance of ARG transcripts. In pig manure, *tet(W)* and *erm(B)* remained highly abundant across all growth stages, whereas the transcript abundances of most other ARGs generally decreased from the juvenile to the adult stage. In parallel, assembly-based analysis was performed for all animal types, followed by CARD annotation of both metagenomic contigs and metatranscriptomic transcripts. This analysis identified ARG subtypes including *msbA*, *novA*, *macB*, *tet(W)*, and *tet(M)*, and their relative abundances across animal types and growth stages are shown in Supplementary Figure 3.

PCoA was conducted to examine the beta diversity of ARG-subtype profiles of various animal types and growth stages using the Bray-Curtis dissimilarity distance calculated at the DNA level (Figure 2c). The two principal coordinates captured 74.2% and 12.5% of the total variations, respectively. Adonis confirmed a statistically significant difference among animal types (R^2^ = 0.75, *p* = 0.001) but not for growth stages (R^2^ = 0.11, *p* = 0.91), indicating that animal type is the primary factor shaping ARGs composition in livestock manure and layer litter.

We examined the beta diversity of ARG-subtype profiles of various animal types and growth stages at the RNA level (Figure 2d). The two principal coordinates captured 64.8% and 17.4% of the total variations, respectively. Adonis analyses confirmed a statistically significant difference among animal types (R^2^ = 0.67, *p* = 0.002) but not for growth stages (R^2^ = 0.20, *p* = 0.62), indicating that the transcriptional activity patterns of ARGs are primarily associated more strongly with animal type than with growth stage.

### 3.3 α-diversity of ARG subtypes and microbial communities

**FIGURE 3:**
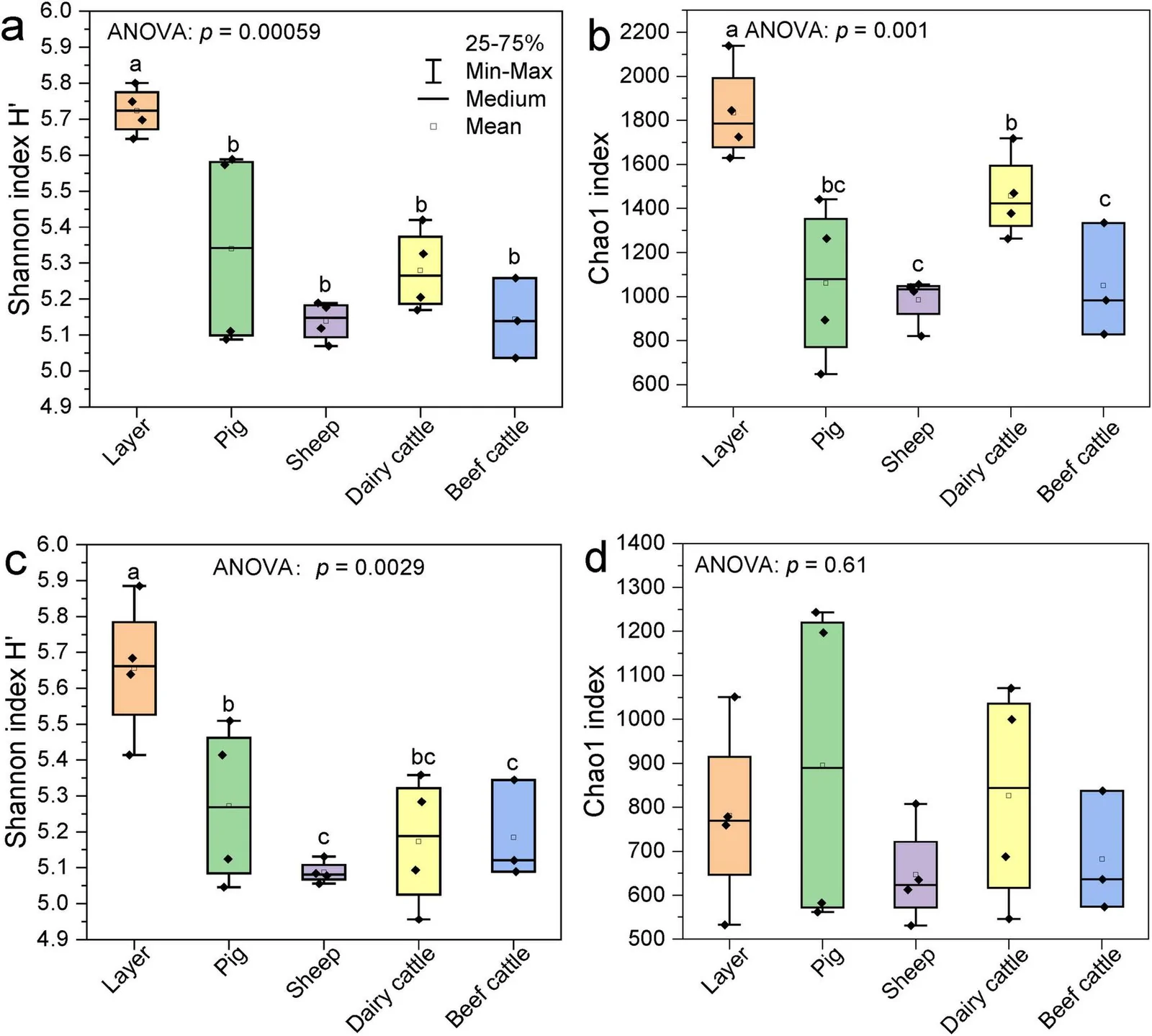
Alpha diversity of ARG subtypes in different livestock manures. **(a)** Shannon index (H’) at the DNA level; **(b)** Chao index at the DNA level; **(c)** Shannon index (H’) at the RNA level; and **(d)** Chao index at the RNA level. Columns marked with different letters indicate significant differences among livestock types (*p* < 0.05) based on ANOVA followed by *post hoc* Tukey’s tests. Each data point represents a sample collected from one animal type at a specific growth stage.

On DNA contigs (Figures 3a, b), ANOVA identified significant differences for animal types on both the Shannon and Chao1 indexes of ARGs. Specifically, layer litter exhibited the highest Shannon index (5.414–5.885) and Chao1 index (532.789–1050.835) among all animal types (*p* = 8.15 × 10^–5^–3 × 10^–3^, *p* = 2 × 10^–4^–0.044). Pig showed the second highest Shannon index (5.046–5.509), which was higher than those observed in ruminants. Ruminants, including sheep, dairy cattle and beef cattle, exhibited comparable Shannon index ranges (5.037–5.420) (Supplementary Table 2). For the Chao1 index, layer (*p* = 2 × 10^–4^–8 × 10^–4^) and dairy cattle (*p* = 0.015–0.044) showed significantly higher values than pig, beef cattle and sheep. These results indicated that layer litter and pig manure had the relatively higher ARG diversity compared to ruminant manures at the DNA contig level.

On RNA transcripts (Figures 3c, d), ANOVA identified significant differences for animal types on the Shannon index but not on the Chao1 index of ARGs. Specifically, layer displayed a significantly higher Shannon index (5.65–5.8) compared to other animal types (*p* = 3.2 × 10^–4^–0.007), reflecting higher transcriptional diversity of ARG subtypes. Pig also showed higher Shannon index (5.089–5.588) than dairy cattle, beef cattle and sheep (*p* = 0.007–0.441) (Supplementary Table 3). However, Chao1 index for five animal types did not differ significantly. Together, these results indicate that the RNA-level ARG diversity differed among animal types, although estimated richness was comparable among groups. even though species richness remained comparable between groups.

At the DNA read level, ANOVA identified significant effect of animal types on the Shannon index and the Chao1 index of microbial community at species level (Supplementary Figures 4a, b). Specifically, sheep exhibited the highest Shannon index (5.434–6.388) and Chao1 index (1343.800–1769.769) followed by layer (Shannon index, 5.136–5.376; Chao1 index, 1302.429–1492.118) (Supplementary Table 4). At the RNA read level, animal type had significant effects on both Shannon and Chao1 indexes (Supplementary Figures 4c, d). Specifically, sheep maintained the highest Shannon index (5.184–6.275) and Chao1 (732–1375) followed by Pig (Shannon index, 4.221–5.393; Chao1 index, 531.667–1466.143) (Supplementary Table 5).

### 3.4 Quantitation of prevalent antibiotic resistance genes

Absolute abundances of 12 target ARGs ranged from 9.39 × 10^5^ to 2.93 × 10^9^ gene copies/g dry weight (Supplementary Figure 5). Among all detected genes, *erm*(B) exhibited the highest absolute abundance of 2.93 × 10^9^ gene copies g^–1^ dry weight. Among the tested *tet* genes, *tet*(X) had the highest concentration of 3.53 × 10^8^ gene copies g^–1^ dry weight. ANOVA showed a significant effect of animal type (*p* = 2.83 × 10^–24^–0.0035) on abundance of *bla*_TEM_ (*p* = 9.07 × 10^–12^), *erm*(B) (*p* = 3.53 × 10^–38^), *qnr*A (*p* = 3.82 × 10^–4^), *sul*1(*p* = 2.05 × 10^–18^), *tet*(Q) (*p* = 7.70 × 10^–30^), and others (Supplementary Table 6). *Post-hoc* Tukey’s test showed that the absolute abundances of *bla*_TEM_, *erm*(B), *erm*(F), *qnr*B, *sul*1, *sul*2, and *tet*(M) were significantly higher in layer litter than in the other animal types (*p* = 6.6 × 10^–15^–3.5 × 10^–3^). The absolute abundances of *tet*(Q), *tet*(W) and *tet*(X) were significantly higher in sheep manure than in the other animals (*p* < 0.05).

**FIGURE 4:**
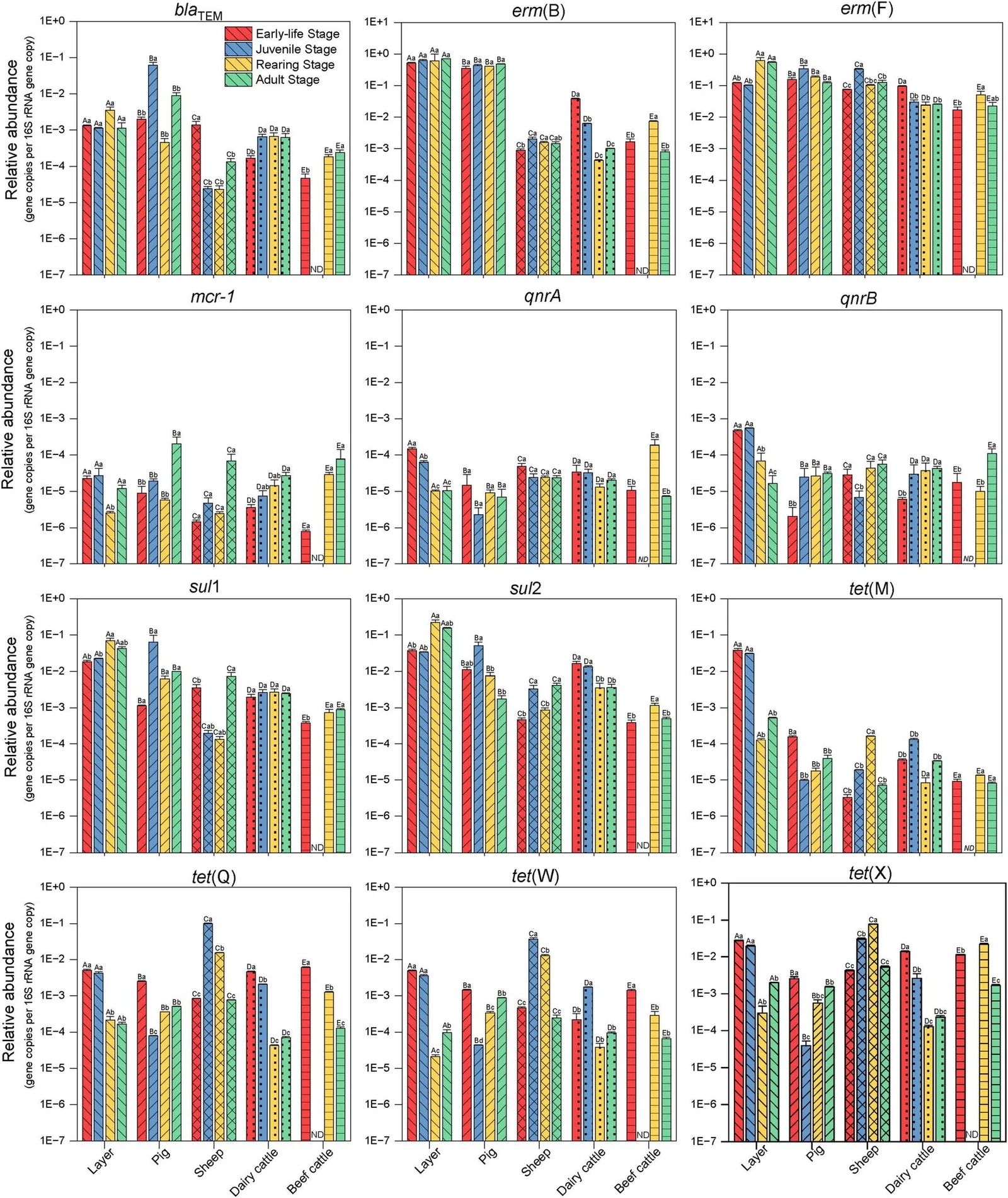
Relative abundance of ARGs in livestock manures. ND represents not determined. Columns marked with different lowercase letters indicate significant differences (*p* < 0.05, *n* = 3 biological replicates) among growth stages within the same livestock type, based on ANOVA followed by *post hoc* Tukey’s tests for multiple comparisons.

When normalized with 16S rRNA gene abundance, *erm*(B) exhibited the highest relative abundance among all detected ARGs, reaching 6.99 × 10^–1^ copies per 16S rRNA gene (Figure 4). *Erm*(B) (6.99 × 10^–1^), *erm*(F) (6.31 × 10^–1^), *qnr*B (5.52 × 10^–4^), *sul*1 (6.96 × 10^–2^), *sul*2 (2.20 × 10^–1^) and *tet*(M) (3.87 × 10^–2^) were predominant in layer, with their abundances being significantly higher than those in the other animals (*p* < 0.05). In addition, *tet*(Q) (9.92 × 1^–2^), *tet*(W) (3.65 × 10^–2^) and *tet*(X) (7.72 × 10^–2^) exhibited highest relative abundances in sheep compared to those in the other animals (*p* < 0.05).

Growth-stage-dependent differences in ARG relative abundance were observed across all five animal types. ANOVA showed that growth stage (*p* = 3.14 × 10^–4^–0.541) had a significant effect on abundance of *bla*_TEM_ (*p* = 6.21 × 10^–4^), *erm*(B) (*p* = 2.81 × 10^–13^), *qnr*A (*p* = 3.54 × 10^–4^), *sul*1(*p* = 5.13 × 10^–8^), and others (Supplementary Table 7). *Post-hoc* Tukey’s test showed that the relative abundances of *bla*_TEM_ and *tet*(Q) were significantly higher (*p* = 0.002–0.009) at the juvenile stage than at the other stages, whereas *tet*(M) was significantly higher (*p* = 0.008–0.035) at the early-life stage than at the other stages.

Overall, qPCR results revealed significant differences in ARG relative abundance across animal types, with the highest abundances observed in layer and sheep. Additionally, growth stage was associated with the abundance patterns of individual ARGs in influencing the abundance patterns of individual ARGs, with the early-life and juvenile stages showing the most significant increases across five animal types.

## 4 Discussion

Integrating metagenomic, metatranscriptomic, and qPCR approaches, our results reveal the composition, temporal dynamics, and transcriptional activity of bacterial communities and ARGs across five types of animals at four growth stages. Our results indicated that the abundance of ARGs in the manure of ruminants was generally lower than that in monogastric animals. In addition, the highest ARG abundance was observed in manure samples from the early-life and juvenile stages indicated by the qPCR results. These findings provide preliminary evidence that may inform the development of feed-management and manure-treatment strategies. However, the effectiveness of such strategies was not directly evaluated in this study.

While metagenomics delineates the genetic potential of ARGs, metatranscriptomics captures their transcriptional activity. Metagenomic analysis identified MLS, tetracycline, and aminoglycoside resistance as the most abundant ARG types across all samples (Supplementary Figure 1), whereas metatranscriptomics revealed that these types were transcriptionally active (Supplementary Figure 1). At the subtype level, *aph(3)-III*, *erm*(B), *lnu*(C), *tet*(Q), and *tet*(W) were identified as highly abundant ARGs across all samples (Supplementary Figure 2), consistent with previous reports showing that *aph(3)-III*, *tet*(Q), and *erm*(B) dominated in swine and poultry manure (Qiu et al., 2022; Yang et al., 2022). Meanwhile, *lnu*(C), *tet*(Q), *tet*(W), and *tet*(40) were transcriptionally active (Supplementary Figure 2), suggesting that these ARGs may serve as key indicators for assessing environmental antibiotic resistance risks. Most previous studies assessing ARGs have relied solely on metagenomics, leaving transcriptionally active ARGs largely unexplored. Our results showed that the transcriptional diversity coverage of ARGs was 97.63% in pig manure, compared with 75.84% in beef cattle, 78.62% in dairy cattle, and 67.40% in sheep manure (Supplementary Table 8). Prior integrated studies have shown that only a subset of ARGs detected on DNA are transcriptionally active (Guitart-Matas et al., 2025; Ma et al., 2022; Wang et al., 2020). For instance, one study reported that 66.5% of ARGs in chickens and 56.6% in pigs are transcriptionally active, with tetracycline, aminoglycoside, and β-lactam resistance genes being the most highly expressed categories (Wang et al., 2020). Another study demonstrated that expression of tetracycline [*tet*(40), *tet*(O), *tet*(W)] and MLS [*erm*(B)] resistance genes is linked to key functions and stability of the active rumen microbiome in beef cattle (Ma et al., 2022). Collectively, these findings highlight that metagenomics alone cannot accurately assess functional antimicrobial resistance risk without considering transcriptional activity.

Both the microbiome and the resistome in manures were strongly associated with animal type. Monogastrics tended to display higher and more variable resistomes than ruminants (Figure 2). Prior studies have indicated that ARG abundance in the rumen and manures of ruminants is generally lower than that in monogastrics (Rothrock et al., 2021). In layers, elevated ARG α-diversity (Figure 3) is consistent with the idea that intensive housing creates repeated opportunities for gut colonization by ARG-carrying taxa and environmental exchange. Notably, source-tracking work in layer systems has shown that environmental reservoirs (e.g., cages and feathers) can contribute substantially to ARG pools (Xiao et al., 2023). For pigs, tetracycline and aminoglycoside resistance genes were the most prominent based on both metagenomic and metatranscriptomic analysis. This result might be associated with antimicrobial usage during pig production where tetracyclines are frequently used for treating or preventing respiratory diseases during post-weaning and fattening stages (Toya et al., 2021). In contrast, ruminants generally exhibited lower ARG richness and abundance, and distinct community structures relative to monogastrics (Figure 2). Rumen-driven ecological filtering as well as antimicrobial exposures and management determine the persistence of ARG hosts (Sabino et al., 2019). Resistomes in cattle production systems vary with management and antimicrobial-use practices (Hitch et al., 2018; Noyes et al., 2016). In sheep, the highest microbial alpha diversity does not necessarily correspond to the highest ARG diversity. Instead, ecological shifts across growth stages such as weaning and diet-driven community restructuring may modulate the resistome by altering the abundance of key ARG-harboring taxa and their associated MGEs (Chai et al., 2024; Zhao et al., 2026). Metagenomic analyses of Awang sheep revealed that dietary and environmental pressures associated with husbandry (grazing versus captivity) significantly reshape the gut resistome, modulating ARG diversity independently of microbial alpha diversity (Zhao et al., 2026).

The qPCR data show that ARG abundances were further modulated by growth stage, with highest absolute and relative ARG abundance during early-life and juvenile stages (Figure 4). Tetracycline and aminoglycoside resistance genes peak at juvenile and rearing stages in pigs, aligning with tetracyclines usage during post-weaning and fattening medication windows (Ma et al., 2023; Toya et al., 2021). In layers, multidrug and tetracycline resistance genes are enriched during the adult stage, reflecting a combination of age-related microbiome succession, persistent early-life effects, and cumulative pressures from production-scale practices and the environment (Ballou et al., 2016; Pan et al., 2024; Ranjitkar et al., 2016; Zhu et al., 2023). By contrast, cattle manure resistomes are comparatively stable across management contexts such as grazing versus confined feeding systems, differences in diet composition, antibiotic use practices, and manure handling or treatment strategies (Hitch et al., 2018; Rothrock et al., 2021; Rovira et al., 2019; Sabino et al., 2019). Taken together, these results indicate that growth stage is associated with variation in the abundance of individual ARGs, although it did not significantly affect overall resistome composition. Growth stage may therefore be considered when interpreting ARG abundance patterns and developing future mitigation strategies.

**FIGURE 5:**
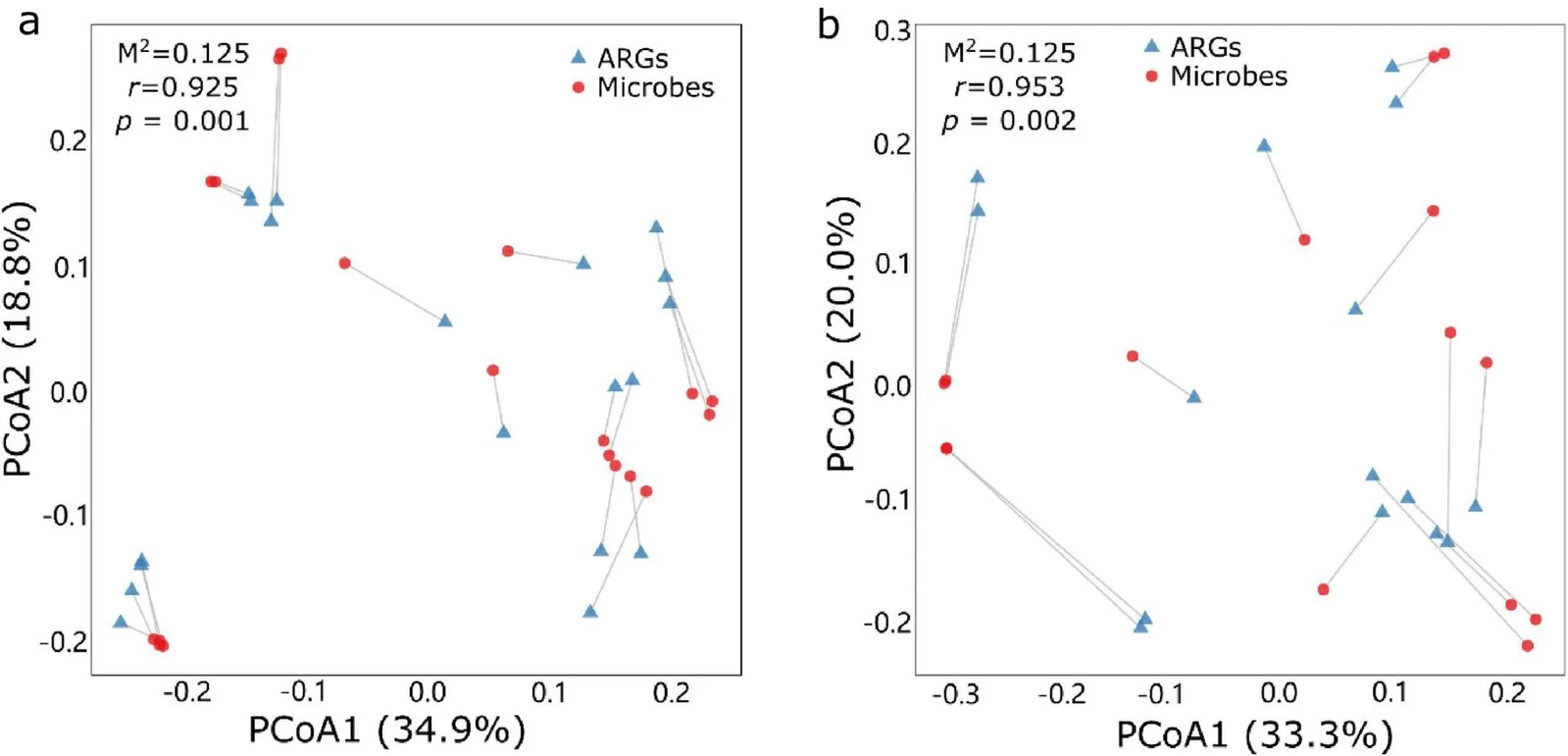
Procrustes analysis revealing the correlations between ARG compositions and microbial community compositions at the DNA **(a)** and RNA levels **(b)**.

Our results indicate that microbiome succession was associated with the observed ARG dynamics at both DNA and RNA level. Procrustes analysis revealed a strong correlation between microbial community and resistome compositions at both DNA (M^2^ = 0.125, *r* = 0.935, *p* = 0.001) (Figure 5a) and RNA level (M^2^ = 0.312, *r* = 0.829, *p* = 0.001) (Figure 5b). The higher M^2^ value at the RNA level (0.312) than at the DNA level (0.125) indicates greater deviation between microbial community and resistome ordinations at the transcriptional level, likely because RNA-level resistome profiles reflect not only the presence of ARGs but also their conditional expression regulated by microbial physiology and environmental pressures. These findings indicated that differences in resistome composition are associated with shifts in microbial compositions. This is consistent with previous studies showing that the abundance of ARGs at the DNA level is influenced by factors such as microbial community composition, niche-specific selective pressures, and host metabolic activities, which regulate the maintenance and activation of ARGs within specific microbial populations and their associated MGEs. In contrast, the active expression of ARGs at the RNA level is regulated by microbial physiological states, host metabolism, and ecological pressures, reflecting the dynamic response of ARG expression to environmental changes and metabolic activity (Liu et al., 2019; Ota et al., 2024; Zhang et al., 2025). Moreover, differences in resistomes between ruminants and monogastrics, with monogastrics displaying higher variability, are likely due to microbial community differences shaped by diet and host physiology, where ruminants have more stable microbial communities, while monogastrics exhibit greater diversity and adaptability, contributing to the observed differences in ARG dynamics. A study found that in dairy cattle manure, both metagenomic and metatranscriptomic data linked ARGs to microbial community composition, with dietary pressures and host metabolism shaping ARG enrichment in specific taxa and their MGEs (Zhang et al., 2024).

This study integrates metatranscriptomics with metagenomics to distinguish between the potential and active resistome, revealing that while 944 ARG subtypes were present, only 579 were actively transcribed. This discrepancy highlights the limitation of DNA-only studies, which cannot differentiate between viable resistance mechanisms and dormant or extracellular DNA. Identifying the 29 ARG subtypes detected at both DNA and RNA levels [e.g., *tet*(W), *sul*1, *erm*(B)] is critical for risk assessment, as their transcription marks an immediate environmental threat. Furthermore, RNA-level data proved more sensitive to physiological shifts across growth stages than taxonomic composition alone. Together with previous studies, these findings suggest potential directions for targeted mitigation in intensive farming systems. Firstly, given the high ARG diversity and abundance observed in layer litter, with *erm*(B) showing a relative higher abundance compared to others at the juvenile stage, optimizing use of antibiotics posing selective pressure may warrant great attentions (Deng et al., 2021). Secondly, several ARGs increased markedly from early-life stage to juvenile stage in pigs; for example, *bla*_TEM_ and *sul*1 showed approximately 40-fold and 71-fold increases, respectively. *tet*(Q) and *tet*(W) in sheep manure increased by approximately 10.5-fold and 7.0-fold, respectively. By integrating these alternatives into stage-specific feeding protocols, it may be possible to reduce ARG proliferation through manure treatment strategies especially for manure collected from growth stage with high ARG abundance and activity (Xu et al., 2023; Kenneth et al., 2023). By implementing stage-specific protocols and focusing on high-activity periods, these approaches may help reduce ARG proliferation, safeguarding environmental and human health.

Several limitations should be acknowledged. First, while the present study identified animal type as main factor influence resistome composition, the differences in diet, housing, antimicrobial exposure, management practices, and manure handling could not be excluded because only one commercial farm was sampled for each animal type, resulting in confounding between animal type effects and differences among farms. Second, growth stages were harmonized across species for comparison but were not necessarily physiologically equivalent, and the design was unbalanced because beef cattle were not sampled at all stages. Third, the limited number of biological replicates and incomplete management and antimicrobial-use records restricted statistical power, mechanistic interpretation, and generalizability. Finally, reliable ARG-OAP read-based metatranscriptomic profiles were unavailable for layer samples. Although the same assembly-based CARD annotation workflow was applied to all animal types and detected transcriptionally represented ARGs in layers, these results were not directly comparable with the read-based estimates from the other animals. Future multi-farm longitudinal studies with larger sample sizes are needed to validate these findings.

## 5 Conclusion

Animal type was identified as the dominant factor influencing microbial community and resistome composition at both the DNA and RNA levels, whereas growth stage was mainly associated with individual ARGs rather than overall resistome structure. In addition, metagenomic and metatranscriptomic analyses detected 944 and 579 ARG subtypes, respectively, but only 29 subtypes were shared between the two levels. Layer litter showed significantly higher number of ARG subtypes than others across tested growth stages based on metagenomics. The significant correlations between microbial community and resistome compositions indicates ARG patterns in livestock manure was determined by microbial community succession. Collectively, the interpretation and future management of livestock manure resistomes should distinguish between broad differences in resistome structure across animal types and variation in the abundance of individual ARGs across growth stages.

## Data Availability

The datasets presented in this study can be found in online repositories. The names of the repository/repositories and accession number(s) can be found below: The sequencing datasets generated and analysed during the current study have been deposited in the NCBI Sequence Read Archive under BioProject accession number PRJNA1456128 and will be made publicly available upon publication.

## References

[B1] Amador P. Fernandes R. Prudêncio C. Duarte I. (2019). Prevalence of antibiotic resistance genes in multidrug-resistant *Enterobacteriaceae* on portuguese livestock manure. *Antibiotics* 8:23. 10.3390/antibiotics8010023 30871244 PMC6466527

[B2] Ballou A. L. Ali R. A. Mendoza M. A. Ellis J. C. Hassan H. M. Croom W. J. et al. (2016). Development of the chick microbiome: How early exposure influences future microbial diversity. *Front. Vet. Sci.* 3:2. 10.3389/fvets.2016.00002 26835461 PMC4718982

[B3] Chai J. Zhuang Y. Cui K. Bi Y. Zhang N. (2024). Metagenomics reveals the temporal dynamics of the rumen resistome and microbiome in goat kids. *Microbiome* 12:14. 10.1186/s40168-023-01733-5 38254181 PMC10801991

[B4] Chalvon-Demersay T. Luise D. Le Floc’h N. Tesseraud S. Lambert W. Bosi P. et al. (2021). Functional amino acids in pigs and chickens: Implication for gut health. *Front. Vet. Sci.* 8:663727. 10.3389/fvets.2021.663727 34113671 PMC8185281

[B5] Chen H. Bai X. Li Y. Jing L. Chen R. Teng Y. (2019). Source identification of antibiotic resistance genes in a peri-urban river using novel crassphage marker genes and metagenomic signatures. *Water Res.* 167:115098. 10.1016/j.watres.2019.115098 31574349

[B6] Dandachi I. Sokhn E. S. Dahdouh E. A. Azar E. El-Bazzal B. Rolain J. M. et al. (2018). Prevalence and characterization of multi-drug-resistant gram-negative bacilli isolated from lebanese poultry: A nationwide study. *Front. Microbiol.* 9:550. 10.3389/fmicb.2018.00550 29628921 PMC5876231

[B7] Deng Z. Hou K. Zhao J. Wang H. (2021). The probiotic properties of lactic acid bacteria and their applications in animal husbandry. *Curr. Microbiol.* 79:22. 10.1007/s00284-021-02722-3 34905106

[B8] Du Y. Gao Y. Hu M. Hou J. Yang L. Wang X. et al. (2023). Colonization and development of the gut microbiome in calves. *J. Anim. Sci. Biotechnol.* 14:46. 10.1186/s40104-023-00856-x 37031166 PMC10082981

[B9] Feinman S. E. Matheson J. C. (1978). *Draft Environmental Impact Statement: Subtherapeutic Antibacterial Agents in Animal Feeds.* Washington, DC: FDA.

[B10] Fu L. Niu B. Zhu Z. Wu S. Li W. (2012). Cd-hit: Accelerated for clustering the next-generation sequencing data. *Bioinformatics* 28 3150–3152. 10.1093/bioinformatics/bts565 23060610 PMC3516142

[B11] Gu Y. Shen S. Han B. Tian X. Yang F. Zhang K. (2020). Family livestock waste: An ignored pollutant resource of antibiotic resistance genes. *Ecotoxicol. Environ. Saf.* 197:110567. 10.1016/j.ecoenv.2020.110567 32289631

[B12] Guitart-Matas J. Vera-Ponce, de León A. Pope P. B. Hvidsten T. R. Fraile L. et al. (2025). Multi-omics surveillance of antimicrobial resistance in the pig gut microbiome. *Anim. Microb.* 7:65. 10.1186/s42523-025-00418-8 40528272 PMC12172226

[B13] He Y. Yuan Q. Mathieu J. Stadler L. Senehi N. Sun R. et al. (2020). Antibiotic resistance genes from livestock waste: Occurrence, dissemination, and treatment. *NPJ Clean Water* 3:4. 10.1038/s41545-020-0051-0

[B14] Hitch T. C. A. Thomas B. J. Friedersdorff J. C. A. Ougham H. Creevey C. J. (2018). Deep sequence analysis reveals the ovine rumen as a reservoir of antibiotic resistance genes. *Environ. Pollut.* 235 571–575. 10.1016/j.envpol.2017.12.067 29331890

[B15] Kadam R. Jo S. Lee J. Khanthong K. Jang H. Park J. (2024). A review on the anaerobic co-digestion of livestock manures in the context of sustainable waste management. *Energies* 17:546. 10.3390/en17030546

[B16] Ke Y. Tang X. Liu C. Liao H. Zhou S. (2026). The cross-kingdom transmission of high-risk antibiotic resistance genes in compost-soil-plant systems and health risks assessment. *Curr. Opin. Environ. Sci. Health* 51:100722. 10.1016/j.coesh.2026.100722

[B17] Kenneth M. J. Koner S. Hsu G. Chen J. Hsu B. (2023). A review on the effects of discharging conventionally treated livestock waste to the environmental resistome. *Environ. Pollut.* 338:122643. 10.1016/j.envpol.2023.122643 37775024

[B18] Li D. Liu C. M. Luo R. Sadakane K. Lam T. W. (2015). Megahit: An ultra-fast single-node solution for large and complex metagenomics assembly via succinct de bruijn graph. *Bioinformatics* 31 1674–1676. 10.1093/bioinformatics/btv033 25609793

[B19] Liu B. Shen Z. Zhou Q. Ren J. Wang H. Zhang D. et al. (2025). Antibiotic resistance genes in the animal manure-amended soil-plant system: Occurrence, transmission, bacterial hosts, and human health risks. *Ecotoxicol. Environ. Saf.* 303:118890. 10.1016/j.ecoenv.2025.118890 40829275

[B20] Liu L. Zhang Q. H. Li M. Z. Li R. T. He Z. Dechesne A. et al. (2025). Single-cell analysis reveals antibiotic affects conjugative transfer by modulating bacterial growth rather than conjugation efficiency. *Environ. Int.* 198:109385. 10.1016/j.envint.2025.109385 40186988

[B21] Liu S. Zhuang Y. Chen T. Gao D. Xiao J. Wang J. et al. (2025). Spatio-temporal characteristics of the gastrointestinal resistome in a cow-to-calf model and its environmental dissemination in a dairy production system. *iMeta* 4:e70047. 10.1002/imt2.70047 40860434 PMC12371271

[B22] Liu Z. Klümper U. Liu Y. Yang Y. Wei Q. Lin J.-G. et al. (2019). Metagenomic and metatranscriptomic analyses reveal activity and hosts of antibiotic resistance genes in activated sludge. *Environ. Int.* 129 208–220. 10.1016/j.envint.2019.05.036 31129497

[B23] Ma L. Song Y. Lyu W. Chen Q. Xiao X. Jin Y. et al. (2023). Longitudinal metagenomic study reveals the dynamics of fecal antibiotic resistome in pigs throughout the lifetime. *Anim. Microbiome* 5:55. 10.1186/s42523-023-00279-z 37941060 PMC10634126

[B24] Ma T. Zaheer R. McAllister T. A. Guo W. Li F. Tu Y. et al. (2022). Expressions of resistome is linked to the key functions and stability of active rumen microbiome. *Anim. Microbiome* 4:38. 10.1186/s42523-022-00189-6 35659381 PMC9167530

[B25] Matheou A. Abousetta A. Pascoe A. P. Papakostopoulos D. Charalambous L. Panagi S. et al. (2025). Antibiotic use in livestock farming: A driver of multidrug resistance? *Microorganisms* 13:779. 10.3390/microorganisms13040779 40284616 PMC12029767

[B26] Mottet A. de Haan C. Falcucci A. Tempio G. Opio C. Gerber P. (2017). Livestock: On our plates or eating at our table? A new analysis of the feed/food debate. *Glob. Food Secur.* 14 1–8. 10.1016/j.gfs.2017.01.001

[B27] Muhammad N. B. Salihu S. Umar A. I. (2020). Comparative of different drying methods on the phytochemical and some nutrient components of scent leaf (*Ocimum gratissimum*). *Asian J. Biochem. Genet. Mol. Biol.* 5 19–23. 10.9734/ajbgmb/2020/v5i330131

[B28] Noyes N. R. Yang X. Linke L. M. Magnuson R. J. Cook S. R. Zaheer R. et al. (2016). Characterization of the resistome in manure, soil and wastewater from dairy and beef production systems. *Sci. Rep.* 6:24645. 10.1038/srep24645 27095377 PMC4837390

[B29] Ota Y. Chen F. Prah I. Mahazu S. Watanabe K. Kinoshita T. et al. (2024). Metatranscriptomic analysis reveals actively expressed antimicrobial-resistant genes and their hosts in hospital wastewater. *Antibiotics* 13:1122. 10.3390/antibiotics13121122 39766512 PMC11672649

[B30] Pan Y. Zeng J. Zhang L. Hu J. Hao H. Zeng Z. et al. (2024). The fate of antibiotics and antibiotic resistance genes in large-scale chicken farm environments: Preliminary view of the performance of national veterinary antimicrobial use reduction action in guangdong, china. *Environ. Int.* 191:108974. 10.1016/j.envint.2024.108974 39186902

[B31] Pham M. N. Nishimura F. Lan J. C. W. Khoo K. S. (2024). Recent advancement of eliminating antibiotic resistance bacteria and antibiotic resistance genes in livestock waste: A review. *Environ. Technol. Innov.* 36:103751. 10.1016/j.eti.2024.103751

[B32] Podhora A. Helming K. Adenäuer L. Heckelei T. Kautto P. Reidsma P. et al. (2013). The policy-relevancy of impact assessment tools: Evaluating nine years of european research funding. *Environ. Sci. Policy* 31 85–95. 10.1016/j.envsci.2013.03.002

[B33] Prunier A. Averos X. Dimitrov I. Edwards S. A. Hillmann E. Holinger M. et al. (2020). Review: Early life predisposing factors for biting in pigs. *Animal* 14 570–587. 10.1017/S1751731119001940 31436143 PMC7026718

[B34] Qiu T. Huo L. Guo Y. Gao M. Wang G. Hu D. et al. (2022). Metagenomic assembly reveals hosts and mobility of common antibiotic resistome in animal manure and commercial compost. *Environ. Microbiome* 17:42. 10.1186/s40793-022-00437-x 35953830 PMC9367140

[B35] Ranjitkar S. Lawley B. Tannock G. Engberg R. M. (2016). Bacterial succession in the broiler gastrointestinal tract. *Appl. Environ. Microbiol.* 82 2399–2410. 10.1128/aem.02549-15 26873323 PMC4959490

[B36] Rothrock M. J. Jr. Min B. R. Castleberry L. Waldrip H. Parker D. Brauer D. et al. (2021). Antibiotic resistance, antimicrobial residues, and bacterial community diversity in pasture-raised poultry, swine, and beef cattle manures. *J. Anim. Sci.* 99:skab144. 10.1093/jas/skab144 33944927 PMC8349186

[B37] Rovira P. McAllister T. Lakin S. M. Cook S. R. Doster E. Noyes N. R. et al. (2019). Characterization of the microbial resistome in conventional and “raised without antibiotics” beef and dairy production systems. *Front. Microbiol.* 10:1980. 10.3389/fmicb.2019.01980 31555225 PMC6736999

[B38] Sabino Y. N. V. Santana M. F. Oyama L. B. Santos F. G. Moreira A. J. S. Huws S. A. et al. (2019). Characterization of antibiotic resistance genes in the species of the rumen microbiota. *Nat. Commun.* 10:5252. 10.1038/s41467-019-13118-0 31748524 PMC6868206

[B39] Sorinolu A. J. Tyagi N. Kumar A. Munir M. (2021). Antibiotic resistance development and human health risks during wastewater reuse and biosolids application in agriculture. *Chemosphere* 265:129032. 10.1016/j.chemosphere.2020.129032 33293048

[B40] Sun Y. Snow D. Walia H. Li X. (2025). Land application of beef cattle manure facilitates the transmission of antibiotic resistance genes from soil to lettuce. *Environ. Sci. Adv.* 4 2127–2137. 10.1039/D5VA00204D

[B41] Sun Y. Staley Z. R. Woodbury B. Riethoven J.-J. Li X. (2024). Composting reduces the risks of resistome in beef cattle manure at the transcriptional level. *Appl. Environ. Microbiol.* 90:e01752-23. 10.1128/aem.01752-23 38445903 PMC11022583

[B42] Toya R. Sasaki Y. Uemura R. Sueyoshi M. (2021). Indications and patterns of antimicrobial use in pig farms in the southern kyushu, japan: Large amounts of tetracyclines used to treat respiratory disease in post-weaning and fattening pigs. *J. Vet. Med. Sci.* 83 322–328. 10.1292/jvms.20-0436 33342965 PMC7972880

[B43] Wang G. Gao X. Cai Y. Li G. Ma R. Yuan J. (2024). Dynamics of antibiotic resistance genes during manure composting: Reduction in herbivores manure and accumulation in carnivores. *Environ. Int.* 190:108900. 10.1016/j.envint.2024.108900 39053194

[B44] Wang H. Qi J.-F. Qin R. Ding K. Graham D. W. Zhu Y.-G. (2023). Intensified livestock farming increases antibiotic resistance genotypes and phenotypes in animal feces. *Commun. Earth Environ.* 4:123. 10.1038/s43247-023-00790-w

[B45] Wang Y. Hu Y. Liu F. Cao J. Lv N. Zhu B. et al. (2020). Integrated metagenomic and metatranscriptomic profiling reveals differentially expressed resistomes in human, chicken, and pig gut microbiomes. *Environ. Int.* 138:105649. 10.1016/j.envint.2020.105649 32200314

[B46] Wood D. E. Salzberg S. L. (2014). Kraken: Ultrafast metagenomic sequence classification using exact alignments. *Genome Biol.* 15:R46. 10.1186/gb-2014-15-3-r46 24580807 PMC4053813

[B47] Xiao S. Mi J. Chen Y. Feng K. Mei L. Liao X. et al. (2023). The abundance and diversity of antibiotic resistance genes in layer chicken ceca is associated with farm environment. *Front. Microbiol.* 14:1177404. 10.3389/fmicb.2023.1177404 37455745 PMC10348872

[B48] Xu Y. Zhu L. Chen S. Wu H. Li R. Li J. et al. (2023). Risk assessment and dissemination mechanism of antibiotic resistance genes in compost. *Environ. Int.* 178:108126. 10.1016/j.envint.2023.108126 37562342

[B49] Yang D. Heederik D. J. J. Scherpenisse P. Van Gompel L. Luiken R. E. C. Wadepohl K. et al. (2022). Antimicrobial resistance genes aph(3’)-iii, erm(b), sul2 and tet(w) abundance in animal faeces, meat, production environments and human faeces in europe. *J. Antimicrob. Chemother.* 77 1883–1893. 10.1093/jac/dkac133 35466367 PMC9244224

[B50] Yang F. Deng B. Liao W. Wang P. Chen P. Wei J. (2019). High rate of multiresistant *Klebsiella pneumoniae* from human and animal origin. *Infect. Drug Resist.* 12 2729–2737. 10.2147/idr.S219155 31564923 PMC6731983

[B51] Yang Y. Jiang X. Chai B. Ma L. Li B. Zhang A. et al. (2016). Args-oap: Online analysis pipeline for antibiotic resistance genes detection from metagenomic data using an integrated structured arg-database. *Bioinformatics* 32 2346–2351. 10.1093/bioinformatics/btw136 27153579

[B52] Yin X. Zheng X. Li L. Zhang A.-N. Jiang X.-T. Zhang T. (2023). Args-oap v3.0: Antibiotic-resistance gene database curation and analysis pipeline optimization. *Engineering* 27 234–241. 10.1016/j.eng.2022.10.011

[B53] Zhang J. Lei H. Huang J. Wong J. W. C. Li B. (2025). Co-occurrence and co-expression of antibiotic, biocide, and metal resistance genes with mobile genetic elements in microbial communities subjected to long-term antibiotic pressure: Novel insights from metagenomics and metatranscriptomics. *J. Hazard Mater.* 489:137559. 10.1016/j.jhazmat.2025.137559 39965334

[B54] Zhang T. Mu Y. Gao Y. Tang Y. Mao S. Liu J. (2024). Fecal microbial gene transfer contributes to the high-grain diet-induced augmentation of aminoglycoside resistance in dairy cattle. *mSystems* 9:e0081023. 10.1128/msystems.00810-23 38085089 PMC10805029

[B55] Zhao E. Li Y. Zhang J. Geng B. (2025). A review on the degradation of antibiotic resistance genes during composting of livestock manure. *Toxics* 13:667. 10.3390/toxics13080667 40863943 PMC12389969

[B56] Zhao S. Wang X. Zhu H. Guo G. Mustafa G. R. Mustafa A. et al. (2026). Metagenomic analysis revealed the distribution of antibiotic resistance genes of awang sheep (*Ovis aries*) gut microbiota. *Front. Microbiol.* 16:1740198. 10.3389/fmicb.2025.1740198 41614136 PMC12847420

[B57] Zhu Y. Pang L. Lai S. Xie X. Zhang H. Yu J. et al. (2023). Deciphering risks of resistomes and pathogens in intensive laying hen production chain. *Sci. Total Environ.* 869:161790. 10.1016/j.scitotenv.2023.161790 36702267

