# Supplementary material for: Animal type outweighs growth stage in shaping the active antibiotic resistome in livestock manure
Source: Front Microbiol. 2026 Sep 9;17:1898413. doi: 10.3389/fmicb.2026.1898413 (PMC13597860; doi:10.3389/fmicb.2026.1898413)
Supplement: Supplementary file 1 [file Supplementary_file_1.docx]

**Supplementary Information**

**Figure S1.** Percentage of ARG types in total abundance at DNA (a) and RNA level (b).

**Figure S2.** Percentage of TOP20 ARG subtypes in total abundance at DNA (a) and RNA level (b).

a

b

**Figure S3.** Relative abundance of ARG subtypes on assembled contigs (a) or transcripts (b). Stage 1, 2, 3, and 4 represents early-life, juvenile, rearing and adult stage, respectively.

**Figure S4.** The α-diversity of microbial communities at species level in livestock manures. (a) Shannon index’H at DNA level; (b) chao index at DNA level; (c) Shannon index’H at RNA level; (d) chao index at RNA level; Column with different letter represent the significant difference (*p* < 0.05) for different livestock types based on ANOVA and post-doc Tukey’ tests.

**Figure S5.** Absolute abundance of ARGs in livestock manures.

**Table S1.** Primers and annealing temperature of ARGs.

| **Target gene** | **Primer** | **Primer sequences (5′–3′)** | **Target size (bp)** | **Annealing temperature (°C)** |
| --- | --- | --- | --- | --- |
| 16s rRNA gene | FW | CGGTGAATACGTTCCCGG | 142 | 57.7 |
|  | RV | GGTTACCTTGTTACGACTT |  |  |
| *bla*_TEM_ | FW | CTTCCTGTTTTTGCTCACC | 580 | 59.8 |
|  | RV | AGCAATAAACCAGCCAGC |  |  |
| *erm*(B) | FW | GGTTGCTCTTGCACACTCAAG | 190 | 59.9 |
|  | RV | CAGTTGACGATATTCTCGATTG |  |  |
| *erm*(F) | FW | TCTGGGAGGTTCCATTGTCC | 340 | 62.8 |
|  | RV | TTCAGGGACAACTTCCAGC |  |  |
| *mcr-1* | FW | CGGTCAGTCCGTTTGTTC | 270 | 61.2 |
|  | RV | CTTGGTCGGTCTGTAGGG |  |  |
| *qnrA* | FW | AGGATTTCTCACGCCAGGATTT | 140 | 62.6 |
|  | RV | CCGCTTTCAATGAAACTGCA |  |  |
| *qnrB* | FW | GCGACGTTCAGTGGTTCAG | 160 | 63.3 |
|  | RV | TGTCCAACTTAACGCCTTGTAA |  |  |
| *sul*1 | FW | CGGCGTGGGCTACCTGAACG | 330 | 68.7 |
|  | RV | GCCGATCGCGTGAAGTTCCG |  |  |
| *sul*2 | FW | GCGCTCAAGGCAGATGGCATT | 260 | 69.0 |
|  | RV | GCGTTTGATACCGGCACCCGT |  |  |
| *tet*(M) | FW | ACAGAAAGCTTATTATATAAC | 160 | 51.9 |
|  | RV | TGGCGTGTCTATGATGTTCAC |  |  |
| *te*t(Q) | FW | AGAATCTGCTGTTTGCCAGT | 160 | 61.5 |
|  | RV | CGGAGTGTCAATGATATTGCA |  |  |
| *tet*(W) | FW | GAGAGCCTGCTATATGCCAGC | 160 | 62.3 |
|  | RV | GGGCGTATCCACAATGTTAAC |  |  |
| *tet*(X) | FW | GAAAGAGACAACGACCGAGAG | 132 | 63.0 |
|  | RV | ACACCCATTGGTAAGGCTAAG |  |  |

**Table S2.** The α-diversity of ARG subtypes at DNA level.

| **Group** | **Sample** | **Shannon** | **Simpson** | **Richness** | **Chao1** |
| --- | --- | --- | --- | --- | --- |
| Layer | Early-life stage | 5.414 | 0.9930 | 412.0 | 532.8 |
|  | Juvenile stage | 5.684 | 0.9940 | 648.0 | 778.2 |
|  | Rearing stage | 5.639 | 0.9940 | 497.0 | 759.4 |
|  | Adult stage | 5.885 | 0.9960 | 568.0 | 1051 |
| Pig | Early-life stage | 5.414 | 0.9910 | 852.0 | 1243 |
|  | Juvenile stage | 5.509 | 0.9920 | 898.0 | 1197 |
|  | Rearing stage | 5.124 | 0.9900 | 506.0 | 582.2 |
|  | Adult stage | 5.046 | 0.9890 | 467.0 | 561.9 |
| Sheep | Early-life stage | 5.131 | 0.9900 | 576.0 | 807.6 |
|  | Juvenile stage | 5.078 | 0.9900 | 502.0 | 635.1 |
|  | Rearing stage | 5.056 | 0.9900 | 419.0 | 530.6 |
|  | Adult stage | 5.084 | 0.9900 | 455.0 | 612.6 |
| Dairy cattle | Early-life stage | 5.358 | 0.9920 | 732.0 | 1071 |
|  | Juvenile stage | 5.284 | 0.9910 | 708.0 | 999.9 |
|  | Rearing stage | 5.093 | 0.9880 | 464.0 | 687.4 |
|  | Adult stage | 4.956 | 0.9880 | 392.0 | 546.2 |
| Beef cattle | Early-life stage | 5.120 | 0.9900 | 438.0 | 636.2 |
|  | Rearing stage | 5.345 | 0.9910 | 538.0 | 837.3 |
|  | Adult stage | 5.089 | 0.9900 | 441.0 | 573.5 |

**Table S3.** The α-diversity of ARG subtypes at RNA level.

| **Group** | **Sample** | **Shannon** | **Simpson** | **Richness** | **Chao1** |
| --- | --- | --- | --- | --- | --- |
| Layer | Early-life stage | 5.749 | 0.9929 | 1487 | 2138 |
|  | Juvenile stage | 5.800 | 0.9933 | 1442 | 1844 |
|  | Rearing stage | 5.646 | 0.9927 | 1186 | 1629 |
|  | Adult stage | 5.698 | 0.9926 | 1245 | 1725 |
| Pig | Early-life stage | 5.574 | 0.9924 | 966.0 | 1442 |
|  | Juvenile stage | 5.588 | 0.9924 | 915.0 | 1264 |
|  | Rearing stage | 5.111 | 0.9891 | 623.0 | 893.4 |
|  | Adult stage | 5.089 | 0.9890 | 553.0 | 648.9 |
| Sheep | Early-life stage | 5.177 | 0.9898 | 769.0 | 1056 |
|  | Juvenile stage | 5.070 | 0.9888 | 639.0 | 820.6 |
|  | Rearing stage | 5.119 | 0.9893 | 735.0 | 1024 |
|  | Adult stage | 5.189 | 0.9898 | 818.0 | 1041 |
| Dairy cattle | Early-life stage | 5.420 | 0.9915 | 1038 | 1717 |
|  | Juvenile stage | 5.326 | 0.9904 | 1089 | 1470 |
|  | Rearing stage | 5.169 | 0.9890 | 876.0 | 1263 |
|  | Adult stage | 5.205 | 0.9895 | 853.0 | 1378 |
| Beef cattle | Early-life stage | 5.037 | 0.9881 | 574.0 | 829.3 |
|  | Rearing stage | 5.259 | 0.9893 | 864.0 | 1335 |
|  | Adult stage | 5.139 | 0.9888 | 774.0 | 983.6 |

**Table S4.** The α-diversity of microbial communities at species levels and at DNA level.

| **Group** | **Sample** | **Shannon** | **Simpson** | **Richness** | **Chao1** | **Pielou** |
| --- | --- | --- | --- | --- | --- | --- |
| Layer | Early-life stage | 5.253 | 0.9830 | 1404 | 1401 | 0.7250 |
|  | Juvenile stage | 5.136 | 0.9800 | 1481 | 1492 | 0.7040 |
|  | Rearing stage | 5.177 | 0.9820 | 1293 | 1302 | 0.7230 |
|  | Adult stage | 5.376 | 0.9880 | 1387 | 1401 | 0.7430 |
| Pig | Early-life stage | 4.302 | 0.9690 | 598.0 | 598.1 | 0.6730 |
|  | Juvenile stage | 4.221 | 0.9660 | 531.0 | 531.7 | 0.6730 |
|  | Rearing stage | 5.393 | 0.9850 | 1451 | 1466 | 0.7410 |
|  | Adult stage | 4.995 | 0.9720 | 1244 | 1251 | 0.7010 |
| Sheep | Early-life stage | 6.388 | 0.9970 | 1760 | 1770 | 0.8550 |
|  | Juvenile stage | 5.434 | 0.9900 | 1386 | 1410 | 0.7510 |
|  | Rearing stage | 5.515 | 0.9880 | 1346 | 1344 | 0.7650 |
|  | Adult stage | 6.125 | 0.9950 | 1633 | 1628 | 0.8280 |
| Dairy cattle | Early-life stage | 5.098 | 0.9870 | 927.0 | 930.6 | 0.7460 |
|  | Juvenile stage | 4.729 | 0.9610 | 1047 | 1051 | 0.6800 |
|  | Rearing stage | 5.473 | 0.9860 | 1025 | 1026 | 0.7890 |
|  | Adult stage | 5.004 | 0.9860 | 872.0 | 872.0 | 0.7390 |
| Beef cattle | Early-life stage | 3.960 | 0.9580 | 388.0 | 393.0 | 0.6640 |
|  | Rearing stage | 3.784 | 0.9450 | 538.0 | 542.0 | 0.6020 |
|  | Adult stage | 4.989 | 0.9840 | 802.0 | 802.0 | 0.7460 |

**Table S5.** The α-diversity of microbial communities at species levels and at RNA level.

| **Group** | **Sample** | **Shannon** | **Simpson** | **Richness** | **Chao1** | **Pielou** |
| --- | --- | --- | --- | --- | --- | --- |
| Layer | Early-life stage | 4.356 | 0.9760 | 244.0 | 244.0 | 0.7920 |
|  | Juvenile stage | 4.672 | 0.9780 | 347.0 | 347.0 | 0.7990 |
|  | Rearing stage | 4.320 | 0.9770 | 188.0 | 188.0 | 0.8250 |
|  | Adult stage | 4.089 | 0.9720 | 136.0 | 136.0 | 0.8320 |
| Pig | Early-life stage | 4.317 | 0.9670 | 634.0 | 637.1 | 0.6690 |
|  | Juvenile stage | 4.448 | 0.9720 | 605.0 | 609.0 | 0.6940 |
|  | Rearing stage | 5.612 | 0.9920 | 955.0 | 955.0 | 0.8180 |
|  | Adult stage | 5.429 | 0.9880 | 855.0 | 856.0 | 0.8040 |
| Sheep | Early-life stage | 6.275 | 0.9970 | 1375 | 1375 | 0.8680 |
|  | Juvenile stage | 5.974 | 0.9950 | 1133 | 1133 | 0.8490 |
|  | Rearing stage | 5.184 | 0.9860 | 732.0 | 732.0 | 0.7860 |
|  | Adult stage | 5.900 | 0.9950 | 932.0 | 932.0 | 0.8630 |
| Dairy  cattle | Early-life stage | 5.171 | 0.9900 | 603.0 | 603.0 | 0.8080 |
|  | Juvenile stage | 5.061 | 0.9890 | 511.0 | 511.0 | 0.8110 |
|  | Rearing stage | 4.349 | 0.9680 | 305.0 | 305.0 | 0.7600 |
|  | Adult stage | 4.729 | 0.9840 | 305.0 | 305.0 | 0.8270 |
| Beef  cattle | Early-life stage | 3.882 | 0.9560 | 222.0 | 222.0 | 0.7180 |
|  | Rearing stage | 4.034 | 0.9730 | 178.0 | 178.0 | 0.7790 |
|  | Adult stage | 5.160 | 0.9890 | 493.0 | 493.0 | 0.8320 |

**Table S6.** ANOVA and post-hoc Tukey’s tests of log_10_-transformed absolute abundance of ARGs based on qPCR results.

| **ARG** | ***p* values** | | |
| --- | --- | --- | --- |
|  | Animal types | Growth stage | Animal types × growth stage |
| 16s rRNA gene | 0.01 | 0.011 | 0.009 |
| *Bla*_TEM_ | 0.003 | 0.005 | 0.01 |
| *erm*(B) | 1.45E-10 | 9.43E-10 | 4.98E-04 |
| *erm*(F) | 0.001 | 0.002 | 0.015 |
| *mcr-1* | 0.004 | 0.006 | 2.71E-06 |
| *qnrA* | 0.008 | 0.01 | 0.257 |
| *qnrB* | 0.004 | 0.005 | 0.013 |
| *Sul*1 | 5.50E-10 | 2.38E-09 | 0.021 |
| *Sul*2 | 4.22E-07 | 1.37E-06 | 0.005 |
| *tet*(M) | 1.28E-10 | 9.43E-10 | 0.041 |
| *tet*(Q) | 0.004 | 0.005 | 1.00E-05 |
| *tet*(W) | 0.013 | 0.013 | 0.003 |
| *tet*(X) | 5.85E-05 | 1.52E-04 | 0.003 |

**Note:** Raw *p*-values are presented. Benjamini–Hochberg FDR correction was applied across the 12 target ARGs, and the adjusted results did not change the statistical conclusions.

**Table S7.** ANOVA and post-hoc Tukey’s tests of log_10_-transformed relative abundance of ARGs based on qPCR results.

| **ARG** | ***p* values** | | |
| --- | --- | --- | --- |
|  | Animal types | Growth stage | Animal types × growth stage |
| *Bla*_TEM_ | 3.36E-06 | 4.48E-06 | 0.77 |
| *erm*(B) | 8.82E-24 | 9.12E-23 | 0.016 |
| *erm*(F) | 1.74E-10 | 5.21E-10 | 0.55 |
| *mcr-1* | 9.69E-05 | 1.16E-04 | 1.33E-06 |
| *qnrA* | 0.144 | 0.144 | 0.112 |
| *qnrB* | 0.002 | 0.003 | 8.59E-04 |
| *Sul*1 | 1.72E-08 | 3.45E-08 | 0.187 |
| *Sul*2 | 8.64E-16 | 3.46E-15 | 0.953 |
| *tet*(M) | 1.52E-23 | 9.12E-23 | 0.333 |
| *tet*(Q) | 3.02E-06 | 4.48E-06 | 6.97E-06 |
| *tet*(W) | 8.46E-09 | 2.03E-08 | 0.02 |
| *tet*(X) | 4.73E-08 | 8.10E-08 | 0.114 |

**Note:** Raw *p*-values are presented. Benjamini–Hochberg FDR correction was applied across the 12 target ARGs, and the adjusted results did not change the statistical conclusions.

**Table S8.** The number of ARG subtypes identified at DNA and RNA levels.

| Sample | Number of ARGs in DNA | Number of ARGs in RNA | Number of Shared ARGs | Shared Ratio (%) |
| --- | --- | --- | --- | --- |
| Pig | 464 | 453 | 369 | 97.63% |
| Sheep | 273 | 184 | 168 | 67.40% |
| Dairy cattle | 449 | 353 | 287 | 78.62% |
| Beef cattle | 269 | 204 | 178 | 75.84% |
